# Paeonol and danshensu combination attenuates apoptosis in myocardial infarcted rats by inhibiting oxidative stress: Roles of Nrf2/HO-1 and PI3K/Akt pathway

**DOI:** 10.1038/srep23693

**Published:** 2016-03-29

**Authors:** Hua Li, Fan Song, Lin-Rui Duan, Juan-Juan Sheng, Yan-Hua Xie, Qian Yang, Ying Chen, Qian-Qian Dong, Bang-Le Zhang, Si-Wang Wang

**Affiliations:** 1 Department of Natural Medicine, School of Pharmacy, Fourth Military Medical University, 169 West Changle Road, Xi’an 710032, People’s Republic of China; 2 Department of Aerospace Physiology, School of Aerospace Medicine, Fourth Military Medical University, 169 West Changle Road, Xi’an 710032, People’s Republic of China; 3 Department of Pharmaceutics, School of Pharmacy, Fourth Military Medical University, 169 West Changle Road, Xi’an 710032, People’s Republic of China; 4 Collaborative Innovation Center for Chinese Medicine in Qinba Moutains, 169 West Changle Road, Xi’an 710032, People’s Republic of China

## Abstract

Paeonol and danshensu is the representative active ingredient of traditional Chinese medicinal herbs Cortex Moutan and Radix Salviae Milthiorrhizae, respectively. Paeonol and danshensu combination (PDSS) has putative cardioprotective effects in treating ischemic heart disease (IHD). However, the evidence for the protective effect is scarce and the pharmacological mechanisms of the combination remain unclear. The present study was designed to investigate the protective effect of PDSS on isoproterenol (ISO)-induced myocardial infarction in rats and to elucidate the potential mechanism. Assays of creatine kinase-MB, cardiac troponin I and T and histopathological analysis revealed PDSS significantly prevented myocardial injury induced by ISO. The ISO-induced profound elevation of oxidative stress was also suppressed by PDSS. TUNEL and caspase-3 activity assay showed that PDSS significantly inhibited apoptosis in myocardia. In exploring the underlying mechanisms of PDSS, we found PDSS enhanced the nuclear translocation of Nrf2 in myocardial injured rats. Furthermore, PDSS increased phosphorylated PI3K and Akt, which may in turn activate antioxidative and antiapoptotic signaling events in rat. These present findings demonstrated that PDSS exerts significant cardioprotective effects against ISO-induced myocardial infarction in rats. The protective effect is, at least partly, via activation of Nrf2/HO-1 signaling and involvement of the PI3K/Akt cell survival signaling pathway.

Ischemic heart disease (IHD) is one of the World’s most threatening diseases because of its high rates of attack, disability and mortality. Myocardial infarction (MI) is a common presentation of IHD. It is the acute condition arising from the critical imbalance between coronary oxygen supply and myocardial demand, resulting in necrosis of the myocardium^1^. This is followed by numerous pathophysiology and biochemical changes such as lipid peroxidation, hyperglycemia, hyperlipidemia, etc.^2^,^3^. A better understanding of the processes involved in MI has stimulated the search for new preventive or therapeutic agents, which could effectively limit and/or treat the myocardial damage.

Medicinal plants, plant extracts and isolated secondary metabolites have traditionally been used to treat several clinical diseases, including oxidative stress-associated diseases^4^, paeonol and danshensu combination thus attracted our attentions. Paeonol (Pae, Fig. 1) is a phenolic acid compound extracted from the famous traditional Chinese medicine (TCM) *Cortex Moutan*. It is well established that paeonol possesses many physiological activities including vascular dilation^5^, prevention of cardiovascular diseases^6^ and improvement of arrhythmia^7^. Danshensu (DSS, Fig. 1) is structurally representative of the water-soluble active components of the well-known traditional Chinese medicine *Radix Salviae Milthiorrhizae*. It has been demonstrated that danshensu can reduce lipid peroxidation on mitochondrial membrane by scavenging free radicals, as well as inhibits permeability and transmission of mitochondrial membrane by reducing thiol oxidation^8^,^9^. We previously found the interesting pharmacokinetic phenomenon that the combination use of paeonol plus danshensu (PDSS) dramatically increased the concentration of danshensu in rat plasma and the concentration of paeonol in rat heart and brain^10^. The pharmacological studies revealed that paeonol and danshensu in combination have synergistic protective effect on diabetes-induced vascular damage in rat, likely through the reduction of oxidative stress and regulation of intracellular Ca^2+ ^11. Actually, in the past few centuries, *Cortex Moutan* and *Radix Salviae Milthiorrhizae*, the original herbal source of paeonol and danshensu, are commonly prescribed in combination, famous as *ShuangDan* prescription (SD), for the prevention or treatment of angina pectoris, myocardial infarction and other IHD symptoms in clinical practice in China^12^,^13^. It is believed that the combined prescribe may increase their preventive or therapeutic effects in treating cardiovascular disease^14^. In our later sequential studies, paeonol and danshensu were proved to be the main active compounds which can be taken as surrogate markers for *ShuangDan* prescription^15^,^16^,^17^,^18^. We also observed the protective effect of paeonol and danshensu combination on antioxidant defense system both in focal cerebral ischemia-reperfusion injured rats and ISO-induced myocardial infarcted rats^19^,^20^ and hypothesized that the protection mechanism of PDSS might be associated with the enhancement of antioxidant system.

However, information on the precise mechanisms of cardioprotective effects of PDSS *in vivo* remains limited. In view of the above facts, the present investigation was undertaken to demonstrate the molecular mechanisms underlying the cardioprotective effect of paeonol combined with danshensu on ISO-induced MI in rats by analysing the activities/expressions of cardiac, apoptotic and antioxidant markers.

## Materials and Methods

### Drugs and chemicals

Paeonol (99.0%, purity) and Danshensu (98.5%, purity) were provided by Dong Ke Madison Bio-tech. Co., Ltd. (Yanglin, Xi’an, China). Isoproterenol hydrochloride was purchased from Tokyo chemical industry Co., Ltd. (Kita-Ku, Tokyo, Japan). Bax, Bcl-2, cytochrome c, TNF-α, Fas, caspase-3, caspase-8, caspase-9, Nrf2, Keap1, HO-1, PI3K, phosphor-PI3K (p-PI3K), Akt and phosphor-Akt (p-Akt) antibodies were purchased from Cell Signaling Technology (Danvers, MA, USA), Goat anti-rabbit, rabbit anti-mouse and anti-rat secondary antibodies were purchased from Kirkegaard & Perry Laboratories, Inc. (Gaithersburg, MD, USA). All other biochemical reagents and chemicals were of analytical grade.

### Animals and Ethics

Male Sprague-Dawley rats (220 ± 20 g) were purchased from Experimental Animal Research Center, the Fourth Military Medical University (Xi’an, China). The animals were maintained in individually ventilated cages (IVC) system (12 h light/dark cycle, 20.3–23.1 °C and 40–50% humidity) during the experiment cycle and fed with standard laboratory food and water ad libitum. There were no significant differences in the body weights of the treated rats when compared with control at the beginning of the study period. The treated rats did not offer any abnormal resistance to drug administration. The treatment schedule did not cause any change in food and water intake pattern. Experimental protocol (2013-0120-R) involving animals was reviewed and approved by the Institutional Animal Care and Use Committee of the Fourth Military Medical University. All experiments were performed in accordance with the relevant guidelines and regulations of the Institutional Animal Care and Use Committee of the Fourth Military Medical University.

### Induction of myocardial injury

Myocardial injury was induced in experimental rats by subcutaneous injection (s.c.) of 85 mg kg^−1^ of ISO daily for 2 consecutive days.

### Experimental Design and Protocol

The experimental animals were divided into five groups of eight rats each.

Group I (Control group): control rats received 0.3% sodium carboxymethyl cellulose (CMC-Na) solution (2 mL kg^−1^ day^−1^, i.g.) for a period of 21 days and normal saline (1 mL kg^−1^, s.c.) on 20th and 21st day.

Group II (ISO group): rats received 0.3% CMC-Na solution for a period of 21 days and ISO (85 mg kg^−1^, s.c.) in normal saline on 20th and 21st day at an interval of 24 h.

Group III (Pae group): rats received paeonol (80 mg kg^−1^ day^−1^, i.g.) for a period of 21 days and ISO on 20th and 21st day.

Group IV (DSS group): rats received danshensu (160 mg kg^−1^ day^−1^, i.g.) for a period of 21 days and ISO on 20th and 21st day.

Group V (PDSS group): rats received paeonol (80 mg kg^−1^ day^−1^, i.g.) and danshensu (160 mg kg^−1^ day^−1^, i.g.) for a period of 21 days and ISO on 20th and 21st day.Paeonol and danshensu were suspended in 0.3% CMC-Na solution.

Control and ISO treated group received equal quantity of vehicle. Dose of Paeonol, danshensu and isoproterenol were selected on the basis of previous studies^20^. At the end of the experimental period (i.e., after 48 h of first dose of ISO administration), rats were anesthetized with pentobarbital sodium (35 mg kg^−1^, i.p.), blood was collected by abdominal aorta and allowed to clot for 1 h at room temperature. Serum was subsequently separated by centrifugation at 4000 rpm for 15 min and stored at −80 °C for biochemical assays. After blood collection, rats were sacrificed by cervical decapitation. Heart tissue was excised and rinsed immediately in ice-cold normal saline, then frozen in liquid nitrogen and stored at −80 °C for further analysis.

### Assay of serum myocardial injury markers

Activity of serum creatine kinase-MB (CK-MB) was measured using commercial kit (Biosino bio-technology and science Inc., Beijing, China). Serum levels of cardiac troponin I (cTnI) and cardiac troponin T (cTnT) were estimated using standard commercial kits (Jiancheng bio-technology and science Inc., Nanjing, China).

### Histopathology

Light microscopic study: Myocardial tissue was excised and fixed in 10% buffered formalin. The fixed tissues were embedded in paraffin, sectioned at 5 μm and stained with hematoxylin and eosin (H&E). The specimens were examined under light microscope (Zeiss Axioskop 40) by the experienced pathologists who were blinded to the experimental protocol. Photomicrographs were taken at ×200 magnification. The histological findings were graded using a scoring system which was classified as: (−) no changes; (+) mild (focal myocytes damage or small multifocal degeneration with slight degree of inflammatory process); (++) moderate (extensive myofibrillar degeneration and/or diffuse inflammatory process); (+++) marked (necrosis with diffuse inflammatory process).

Transmission electron microscopy: Small pieces of myocardial tissue were retrimmed into 1mm^3^ blocks and fixed in 4% glutaraldehyde overnight. The next day, the blocks were washed trice with 0.1 M phosphate buffer and post-fixed for 2 h in 1% osmium tetraoxide in the same buffer at 4 °C. After several washes in 0.1 M phosphate buffer, the specimens were dehydrated using graded acetone solutions and embedded in Spon812. Semi-thin (1 mm) as well as ultrathin sections (50 nm) were cut by an ultramicrotome (LKB-Nova, Sweden). The sections were stained in alcohol uranyl acetate and lead citrate and viewed under JEM-2000EX transmission electron microscope (Hitachi Co., Ltd., Japan).

### Assay of myocardial reactive oxygen species (ROS) level, thiobarbituric acid reactive substances (TBARS) level and reduced glutathione/oxidized glutathione (GSH/GSSG) ratio

Tissue ROS was measured as described by Shinomol^21^, based on the 2′7′-dichlorodihydrofluorescein diacetate (H_2_DCFH-DA), a non-polar compound that, after conversion to a polar derivative by intracellular esterases, can rapidly react with ROS to form the highly fluorescent compound dichlorofluorescein (DCF). Peroxide level in heart was determined as thiobarbituric acid reactive substances (TBARS) using a reagent kit (BioAssay Systems, CA, USA). Ratio of glutathione to oxidized glutathione (GSH/GSSG) was determined and calculated according to the protocols of assay kit (Jiancheng bio-technology and science Inc., Nanjing, China). Data are expressed as pmol DCF/min/mg protein for ROS level and μmol malondialdehyde (MDA) equivalents for TBARS level, respectively.

### Terminal deoxynucleotidyl transferase-mediated dUTP nick end-labeling (TUNEL) assay

The 5-μm-thick paraffin sections of rat myocardial tissues were prepared and the apoptotic myocardial cells were detected with a TUNEL assay kit (fluorescein *in situ* cell death detection kit; Roche, Mannheim, Germany) according to the manufacturer’s instructions. 4′-6-Diamidino-2-phenylindole (DAPI) was used as a counter staining. The apoptotic cells were analyzed by fluoresce microscopy (Nikon, Eclipse TI-SR). The number of TUNEL positive nuclei was calculated by Image-Pro Plus 6.0 software (Media Cybernetics Inc, Silver Spring, MD, USA).

### Caspase-3 activity assay

Cardiac caspase-3 activity was measured by using caspase-3 activity assay kits (Beyotime institute of biotechnology, Shanghai, China). Myocardial tissue was homogenized in ice-cold lysis buffer. The homogenates were centrifuged for 15 min at 16,000 rpm at 4 °C. Supernatants were collected and a volume of 10 μL was incubated with 10 μL caspase-3 substrate acetyl-Asp-Glu-Val-Asp *p*-nitroanilide (AcDEVD-*p*NA) at 37 °C for 1.5 h. The colorimetric release of *p*NA (*p*-nitroaniline) from the AcDEVD-*p*NA substrate was record at 405 nm. Caspase-3 activity was expressed as unit/h/mg protein.

### Assay of NQO1, GST and GCL activity

Activities of NAD(P)H: quinine oxidoreductase 1 (NQO1) was measured as the dicumarol-inhibitable reduction of dichlorophenol indophenol (DCIP) according to the procedures of Zhu *et al.*^22^. Glutathione-S-transferase (GST) was measured according to the procedures described previously^20^. 1-Chloro-2, 3-dinitrobenzene (CDNB) was used as the substrate for GST. γ-Glutamylcysteine ligase (GCL) activity was determined at 25 °C spectrophotometrically using a commercial kit (Kemin biotechnology, Suzhou, China). Data are expressed as nmol of DCIP reduced/min/mg protein for NQO1 activity, μmol of CDNB conjugated/min/mg protein for GST activity and μmol of NADPH oxidized/min/mg protein for GCL activity, respectively.

### Western Blot Analysis

Radioimmunoprecipitation (RIPA) buffer (Guge Biotechnology, Wuhan, China) and nuclear and cytoplasmic extraction kit (Guge Biotechnology, Wuhan, China) containing 1% protease inhibitor assay cocktail (Guge Biotechnology, Wuhan, China) were used to protein extract, respectively, from myocardial tissue. Nuclear fraction was used for Nrf2 and cytosolic fraction was used for Nrf2 and HO-1 immunoblotting. In total protein extract, protein expressions of Bax, Bcl-2, caspase-3, caspase-8, caspase-9, cytochrome c, TNF-α, Fas, Keap1, PI3K, p-PI3K, Akt and p-Akt were determined. The protein concentration was estimated using BCA reagent (Bio-Rad Laboratories, Inc., Hercules, CA, USA), and equal amounts of the proteins (40 μg) were separated on 10% SDS–PAGE and transferred to polyvinylidine difluoride (PVDF) membrane (Millipore). Membranes were blocked in 5% nonfat dry milk (Yili, Inc., Inner Mongolia, China) in TBST buffer for 1 h at room temperature and then incubated with primary antibodies such as Bax, Bcl-2, caspase-9, caspase-8, caspase-3, Fas, TNF-α, cytochrome c, HO-1, Nrf2, Keap1, PI3K, p-PI3K, Akt and p-Akt with gentle shaking overnight at 4 °C. β-actin (Santa Cruz Biotechnology, CA, USA) and Lamin B (Bioworld Technology, MN, USA) were used as internal control for equal loading of total and nuclear protein extract, respectively. After this, membranes were incubated with their corresponding horseradish peroxidase (HRP)-conjugated secondary antibodies for 2 h at room temperature and then washed thrice with TBST for 15 min. Protein bands were visualized by enhanced chemiluminescence method using ECL kit (Guge Biotechnology, Wuhan, China). The densitometry analysis was performed by AlphaEaseFC software (Alpha Innotech, San Leandro, CA, USA).

### Statistical analysis

Results are shown as mean ± S.D. One-way ANOVA was carried out, and the statistical comparisons among the groups were performed with Tukey’s test using GraphPad Prism 6.0 statistical package program (GraphPad Software, Inc., La Jolla, CA, USA). P-values less than 0.05 were considered as statistically significant.

## Results

### PDSS decreases level of myocardial injury markers in the serum

**Figure 2:**
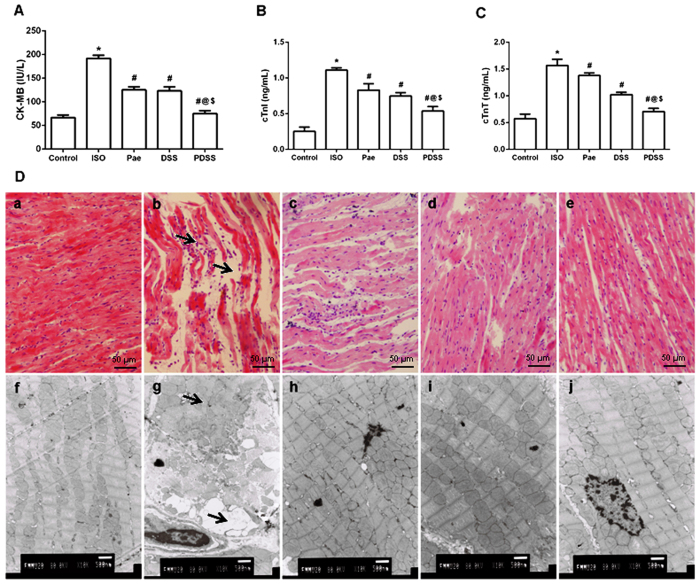
Effect of PDSS on ISO-induced changes in serum-specific cardiac injury biomarkers [(**A**) CK-MB, (**B)** cTnI, **(C)** cTnT; values are expressed as mean ± S.D. (n = 8). ^*^P < 0.001 vs. Control; ^#^*P* < 0.001 vs. ISO; ^@^*P* < 0.001 vs. Pae; ^$^*P* < 0.001 vs. DSS (one-way ANOVA).] and on ISO-induced histopathologic changes of heart [(**D)** (a) control, (b) ISO, (c) Pae, (d) DSS and (e) PDSS; (a–e) belongs to light microscopic study; heart tissues were stained with hematoxylin and eosin and visualized under light microscope at ×200 magnification. (**D)** (f) control, (g) ISO, (h) Pae, (i) DSS and (j) PDSS; (f–j) belongs to TEM study; heart tissues were stained in alcohol uranyl acetate and lead citrate and viewed under transmission electron microscope at ×10000 magnification].

Administration of ISO significantly (P < 0.001) increased the serum activitiy/levels of CK-MB, cTnT and cTnI (Fig. 2A–C) in rats. The three aforesaid indices were obviously lowered either by paeonol alone or by danshensu alone in ISO-administered rats. Pretreatment of paeonol and danshensu combination showed a significant (P < 0.001) decrease in the activitiy/levels of serum myocardial injury markers when compared to ISO, Pae or DSS groups.

### PDSS alleviates ISO-induced histological changes in the myocardial tissue

In line with the manifested serum marker enzymes changes, histopathological observations of ISO-induced rats revealed marked necrotic changes in myofibrils with intense infiltration of neutrophil granulocytes and interstitial edema (Fig. 2D-b). Tissue sections from paeonol or danshensu treatment rats showed mild infiltration with neutrophil granulocytes and discontinuity with adjacent myofibrils but the morphology of cardiac muscle fibers was relatively well preserved with mild focal necrosis when compared to ISO-induced group. PDSS group showed better cardioprotection as observed by the absence of adverse pathological changes in the cardiac tissue (Fig. 2D-e). (See the Supplementary Information for the scoring table).

Electron micrographs of ISO-induced rat hearts show extensive loss of cell membrane integrity, myonecrosis along with significant disruption of myofilaments, the appearance of vacuoles within the cell, disarrangement of cristae and changes in the mitochondrial architecture (Fig. 2D-g). On the other hand, occasional disruption of myofilaments, mild interstitial edema and less accumulation of electron dense material in mitochondria were noticed in paeonol or danshensu alone treated rats. PDSS group showed a better morphology than individual treatment groups, myocardial ultrastructure was found to be well preserved and less evidence of myocyte injury was observed in the cardiac tissue (Fig. 2 D-j).

### PDSS mitigates lipid peroxidation in the myocardial tissue

**Figure 3:**
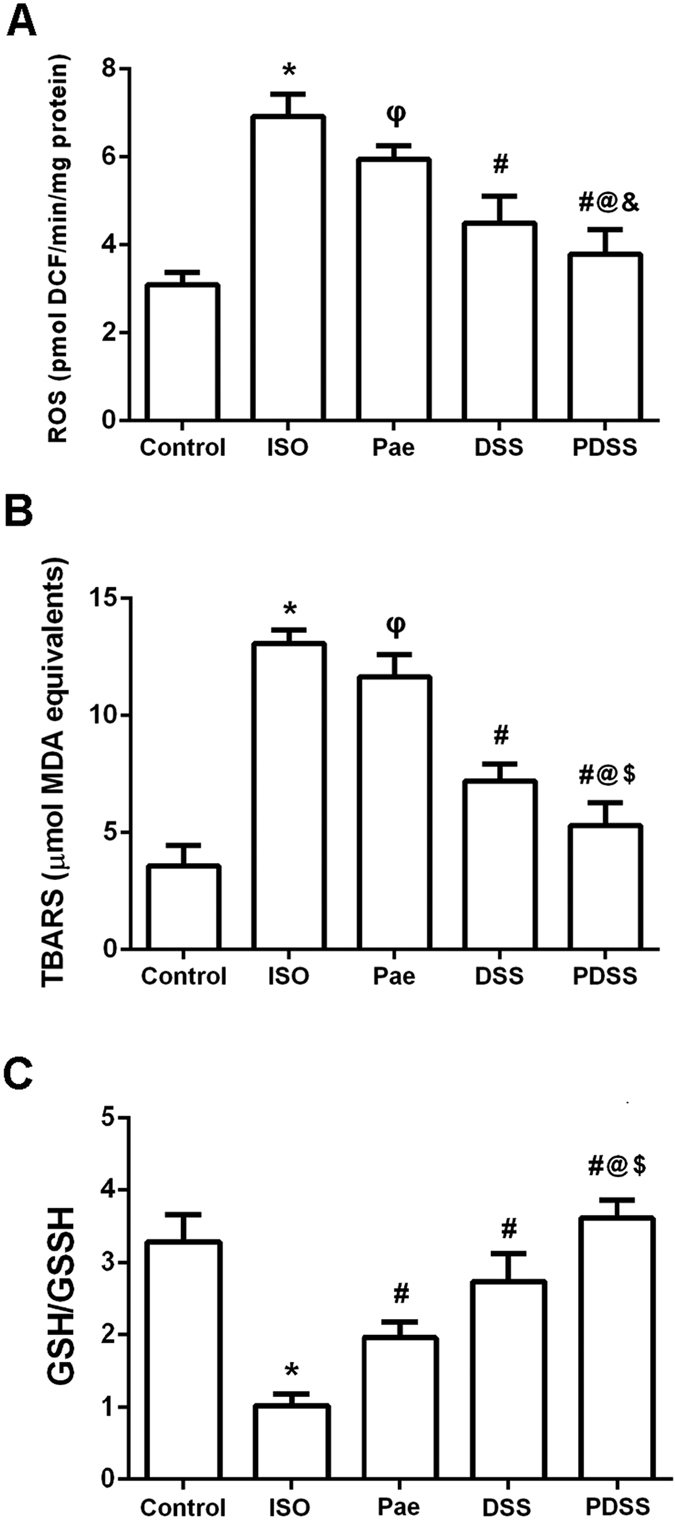
Effect of PDSS on ISO-induced oxidative damage in myocardial. (**A)** reactive oxygen species (ROS) level, (**B)** thiobarbituric acidreactive substances (TBARS) level and (**C)** reduced glutathione/oxidized glutathione (GSH/GSSG) ratio. Values are expressed as mean ± S.D. (n = 8). ^*^P < 0.001 vs. Control; ^φ^*P* < 0.05 vs. ISO; ^ε^*P* < 0.01 vs. ISO; ^#^*P* < 0.001 vs. ISO; ^@^*P* < 0.001 vs. Pae; ^$^*P* < 0.001 vs. DSS (one-way ANOVA).

The myocardial ROS, TBARS and GSH/GSSG ratio are shown in Fig. 3, respectively. Myocardial ROS and TBARS, which indicates the extent of lipid peroxidation, was significantly (P < 0.001) increased in the heart tissue of ISO group when compared to normal control group, whereas, a significant (P < 0.001) decrease in GSH/GSSG ratio (Fig. 3C) were observed in the ISO-induced rats. Paeonol and danshensu significantly decreased the levels of ROS and TBARS and significantly increased the GSH/GSSG ratio when compared to ISO-induced rats. Combination of paeonol and danshensu showed significant (P < 0.05 or P < 0.001) protection than individual treatment groups in mitigating the parameters of ROS, TBARS and GSH.

### PDSS reduces Keap1 expression while increases Nrf2 nuclear accumulation and elevates activities of the phase II enzymes

**Figure 4:**
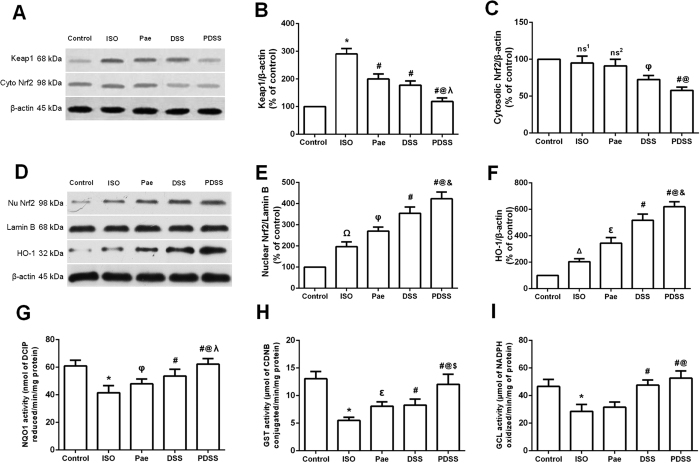
Effect of PDSS on ISO-induced changes in myocardial nuclear translocation of nuclear factor erythroid 2-related factor 2 (Nrf2) and activities of the phase II enzymes. Graphs represents the (**A)** Immunoblot of myocardial Keap1 and cytosolic Nrf2 protein expressions; densitometry analysis of (**B)** Keap1 and (**C)** cytosolic Nrf2 protein expressions; **(D)** Immunoblot of myocardial nuclear Nrf2 and heme oxygenase-1 (HO-1) protein expressions; densitometry analysis of (**E)** nuclear Nrf2 and (**F)** HO-1 protein expressions; myocardial **G** NAD(P)H:quinine oxidoreductase 1 (NQO1), (**H)** glutathione-S-transferase (GST) and (**I)** γ-glutamylcysteine ligase (GCL) activities. Values are expressed as mean ± S.D. (n = 3 in immunoblot and n = 8 in assay of phase II enzymes activities, respectively). ns^1^–non significant vs. Control, ^Δ^*P* < 0.05 vs. Control, ^Ω^*P* < 0.01 vs. Control; ns^2^–non significant vs. ISO, ^φ^*P* < 0.05 vs. ISO, ^ε^*P* < 0.01 vs. ISO, ^#^*P* < 0.001 vs. ISO; ^@^*P* < 0.001 vs. Pae; ^&^*P* < 0.05 vs. DSS, ^λ^*P* < 0.01 vs. DSS, ^$^*P* < 0.001 vs. DSS (one-way ANOVA).

We measured protein levels of Keap1, Nrf2 and HO-1, and activities of NQO1, GST and GCL to evaluate whether paeonol and danshensu combination was able to activate the Nrf2 antioxidant signaling pathway in myocardial tissues (Fig. 4). As shown in Fig. 4A,B, the expression of Keap1 was significantly elevated in the myocardial tissues of ISO-induced group. The altered protein expressions were normalized in paeonol and danshensu groups and, to a greater extent, in PDSS group. Treatment with ISO slightly reduced cytosol Nrf2 levels, and the changes were further promoted in paeonol, danshensu and PDSS groups (Fig. 4A,C). Conversely, ISO-induced rats showed mild increase in nuclear accumulation of Nrf2, whereas paeonol treatment resulted in a significant increase in accumulation of Nrf2 in the nuclear fraction as compared with ISO-induced rats, although a much higher increase (P < 0.001) was observed in both danshensu and PDSS group (Fig. 4D,E). Similarly, as shown in Fig. 4F, HO-1 expression was significant increased by paeonol group and, to a greater extent, by danshensu and PDSS group. In addition, a marked increase in heart NQO1and GST activities were observed in both paeonol and danshensu or PDSS group (Fig.4G,H). The activity of NQO1, and GST in the PDSS group was significantly (P < 0.001) higher than in all other groups. However, it seems that paeonol alone did not affect GCL activity whereas GCL activity was significantly increased in the danshensu and PDSS groups (Fig. 4I).

### PDSS inhibits ISO-induced apoptosis in the myocardial tissue

**Figure 5:**
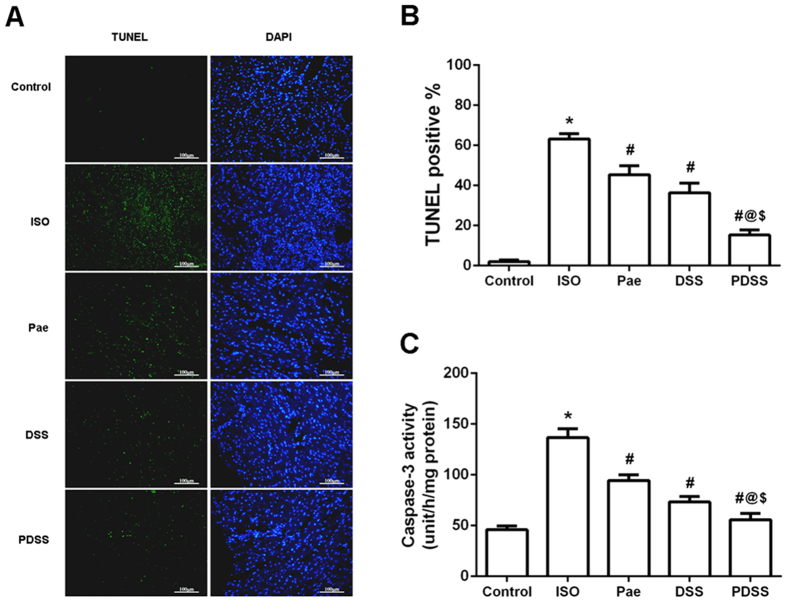
Effect of PDSS on ISO-induced myocardial apoptosis *in vivo*. (**A)** Representative photographs of TUNEL staining in different groups. Myocardial tissues were stained and analyzed with TUNEL (green) and counterstained with DAPI (blue). (**B)** Quantitative analysis of TUNEL positive cells. The percentage of TUNEL positive cells was expressed as percent DAPI stained cells. (**C)** Myocardial caspase-3 activity. Values are expressed as mean ± S.D. (n = 3 in TUNEL staining and n = 8 in assay of caspase-3 activity, respectively). ^*^P < 0.001 vs. Control; ^#^*P* < 0.001 vs. ISO; ^@^*P* < 0.001 vs. Pae; ^$^*P* < 0.001 vs. DSS (one-way ANOVA).

To determine the features of ISO-induced myocardial cell death, TUNEL and caspase-3 activity assay were carried out. Representative photomicrographs of *in situ* detection of DNA fragments in heart tissue are shown in Fig. 5A. Total nuclei were labeled with DAPI (blue), and apoptotic nuclei were detected by TUNEL staining (green). The summary of percent TUNEL-positive myocytes is shown in Fig. 5B. The number of TUNEL-positive nuclei stain was significantly increased in ISO-induced rats (63.25 ± 2.53%) when compared to normal control rats (2.04 ± 0.80%). Treatment of paeonol and danshensu alone or in combination resulted in a reduction (P < 0.001) in the percentage of TUNEL-positive cells, 45.42 ± 4.50%, 36.36 ± 4.72% and 15.32 ± 2.52%, respectively. There were significant differences in the percentage between the alone treated group and the PDSS group (P < 0.001). The executioner caspase of apoptosis, like caspase-3 activities (Fig.5C) was also significantly (P < 0.001) increased in ISO-induced group compared to normal control group. Whereas, pretreatment with paeonol, danshensu and PDSS followed by ISO significantly (P < 0.001) decreased the caspases-3 activity as compared to ISO-induced rats.

### PDSS modulates ISO-induced expression of apoptotic proteins in the myocardial tissue

**Figure 6:**
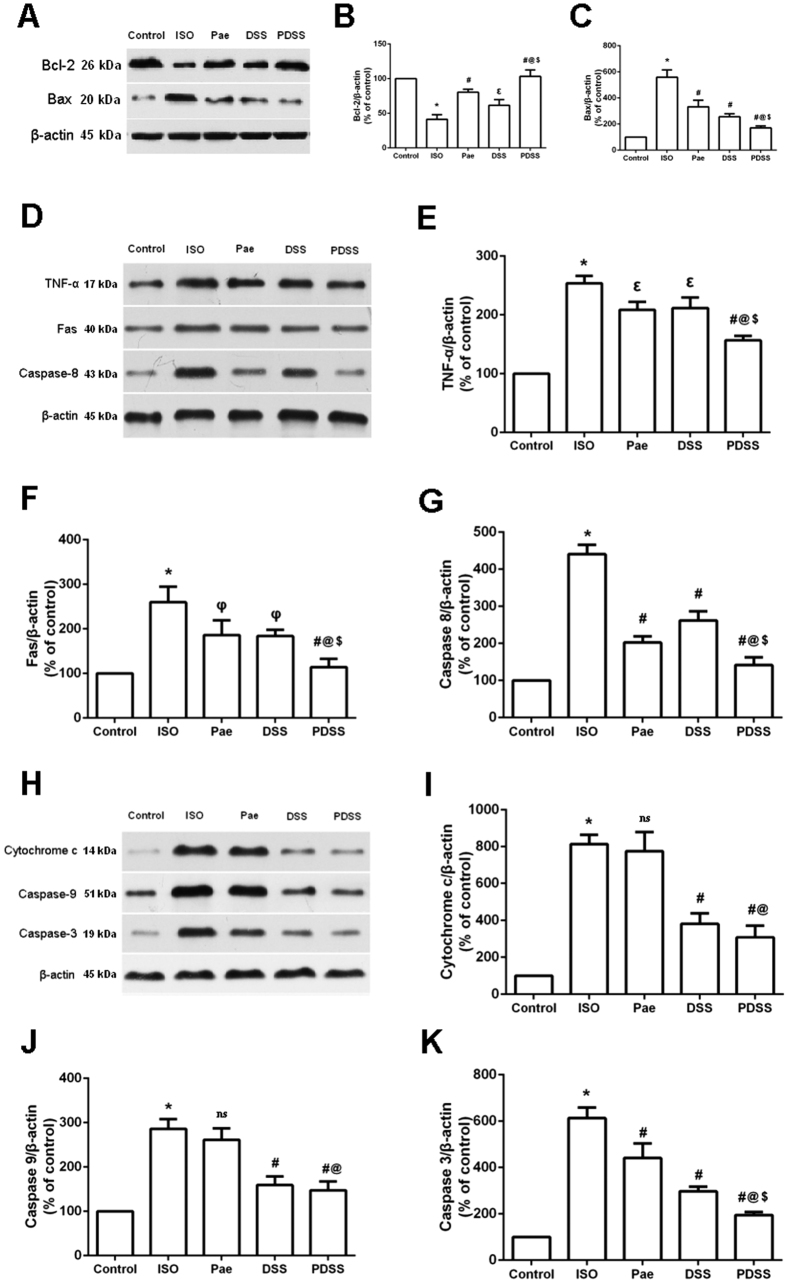
Effect of PDSS on ISO-induced apoptosis-related protein expression in myocardical tissue. Graphs represents immunoblot of (**A)** Bcl-2 and Bax, (**D)** TNF-α, Fas and caspase-8, (**H)** cytochrome c, caspase-9 and caspase-3; and densitometry analysis of (**B)** Bcl-2, **(C)** Bax, (**E)** TNF-α, (**F)** Fas, (**G)** caspase-8, (**I)** cytochrome c, (**J)** caspase-9 and (**K)** caspase-3, respectively. Values are expressed as mean ± S.D. (n = 3).^*^P < 0.001 vs. Control; ns–non significant vs. ISO, ^φ^*P* < 0.05 vs. ISO, ^ε^*P* < 0.01 vs. ISO, ^#^*P* < 0.001 vs. ISO; ^@^*P* < 0.001 vs. Pae; ^$^*P* < 0.001 vs. DSS (one-way ANOVA).

To assess the effect of PDSS on ISO-induced myocardial apoptosis, we investigated the protein expression of apoptosis signaling molecules such as Bax, Bcl-2, caspase-3, caspase-8, caspase-9, cytochrome c, Fas and TNF-α in myocardial tissue of different experimental groups. As shown in Fig. 6A–C, ISO-induced rats showed markedly down regulated protein expression of Bcl-2 and increased Bax expression in myocardial tissue. Pretreatment with paeonol and danshensu alone or in combination significantly upregulated protein expression of Bcl-2 and attenuated expression of Bax compared to ISO-induced rats. Moreover, There were significantly (P < 0.001) difference between the alone treated group and PDSS group. Figure 6D–G depicts the protein expression of TNF-α, Fas and caspase-8 in heart tissue of control and experimental rats. Expression of TNF-α, Fas and caspase-8 were increased significantly in ISO-rats and were reduced significantly upon paeonol, danshensu and PDSS administration. Danshensu alone caused a much smaller decrease in caspase-8 expression as compared with the paeonol and PDSS group, although the decrease was significant compared to the ISO-induced rats. In addition, as represented in Fig. 6H–K, ISO administration also caused a significant upregulation of cytochrome c, caspase-9 and caspase-3 expressions in myocardial tissue. Pretreatment with paeonol alone did not affect cytochrome c and caspase-9 expression, whereas the expression of cytochrome c and caspase-9 in danshensu alone group and PDSS groups were significant down-regulated as compared to ISO-induced group, respectively. Caspase-3 expression was attenuated by paeonol and, to a greater extent, by danshensu. Although there is a much more significant decrease of caspase-3 expression in the PDSS group than the individual treatment groups.

### PDSS-mediated protective action involves PI3K/Akt activation

**Figure 7:**
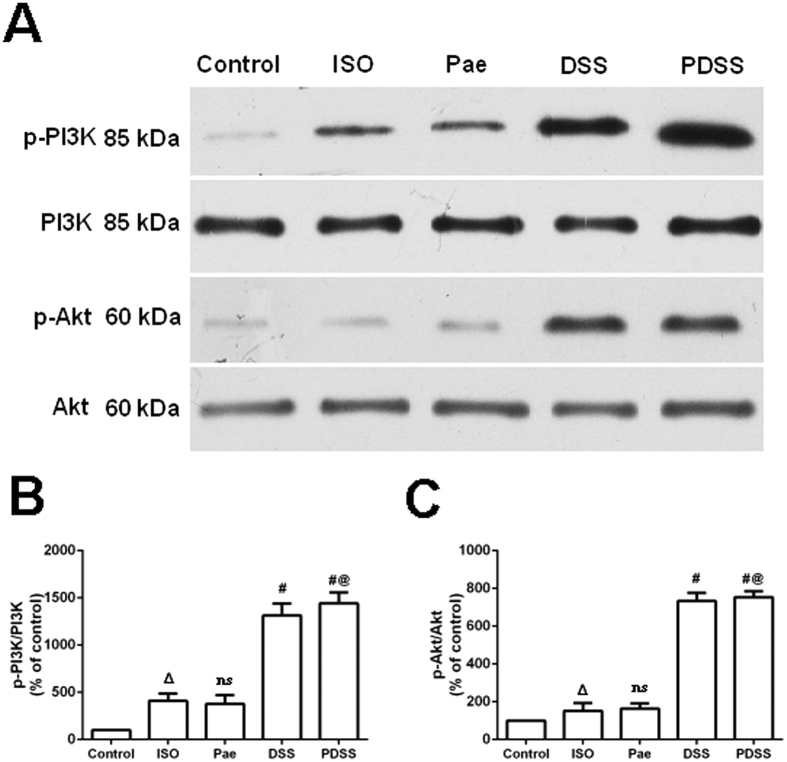
Involvement of the PI3K/Akt activation in cardioprotection of PDSS on ISO-induced myocardial injure. (**A**) Immunoblot and (**B**,**C**) densitometry analysis showing the protein expression of **(B)** p-PI3K, PI3K, **(C)** p-Akt and Akt in myocardial tissues of the control and experimental animals. Values are expressed as mean ± S.D. (n = 3). ^Δ^*P* < 0.05 vs. Control; ns–non significant vs. ISO, ^#^*P* < 0.001 vs. ISO; ^@^*P* < 0.001 vs. Pae (one-way ANOVA).

In order to determine whether the PI3K/Akt pathways were activated after administration of paeonol and danshensu in ISO-induced rats, the protein levels of PI3K and Akt in myocardial tissues were evaluated by western blot analysis. As shown in Fig. 7A–C, paeonol caused only a marginal increase in phosphorylated PI3K/Akt which is no significant difference compared to ISO-induced group. In contrast, significant increase in phosphorylated PI3K/Akt was observed in danshensu and PDSS treated rats. Thus it is plausible that Akt activation is involved in the protective effect of PDSS but it could not entirely explain the protective effect, especially of paeonol.

## Discussion

Traditional Chinese medicines (TCMs) and their ingredients have been used as the most important therapeutic agents in China from times immemorial. In clinical practice, TCMs are commonly prescribed in combination form which usually consisted of dozens of chemical components from several herbs^15^. It invokes the conception of system-based intervention strategies which use multi-inputs to solve the complexity of a disease state^23^. As a result, the combinatorial use reinforces the overall effects or eliminates the possible adverse reactions of certain drugs. Paeonol and danshensu, the two most well-known active ingredients in *Cortex Moutan* and *Radix Salvia Miltiorrhizae*, respectively, have been validated to producing promising therapeutic effects in combination form on cerebrovascular and cardiovascular diseases animal models in our previous pharmacological studies^19^,^20^. Despite those pharmacological benefits, the molecular mechanisms by which paeonol and danshensu combination elicits cardioprotective effects are still unclear. The results from the current study characterized the cardioprotective properties of paeonol and danshensu, furthermore, provided evidences that these cardioprotective effects were in part mediated through activation of Nrf2 and PI3K/Akt signaling.

Over-release of catecholamine is a principal factor related to myocardial injure in many cardiovascular diseases, such as myocardial ischemia, hypertrophy, and heart failure. Isoproterenol is a β-adrenergic agonist that in excess doses causes severe stress in myocardium, resulting in subendocardial myocardial ischemia, hypoxia and necrosis^24^,^25^. Number of studies reported that the pathophysiological, morphological and metabolic changes occur in the heart of experimental animals following ISO administration resembled those produced by myocardial ischemia in human beings^25^,^26^,^27^. ISO-induced myocardial injury involves membrane permeability alterations that bring about loss of function and integrity of myocardial membranes. In the present study, increased serum specific cardiac biomarkers levels such as CK-MB, cardiac troponin I and cardiac troponin T with abnormal histoarchitecture of heart tissue were observed in the ISO-induced rats, indicating the leakage and loss of functional integrity and/or necrotic damage of cell membrane. Pretreatment with paeonol and danshensu combination significantly decreased not only the serum levels of CK-MB, cardiac troponin I and cardiac troponin T but also the pathological changes such as loss of myofibrillar alignment, myocardial degeneration, severe cytoplasmic vacuolization and infiltration of inflammatory cells compared ISO-induced rats (Fig. 2). These primary findings confirmed our previous study, in which PDSS regulated the ECG and hemodynamic parameter in MI rats, and further indicated the cardioprotective effect of combined use of paeonol and danshensu^20^,^28^.

Accumulating evidence has also shown that ISO causes oxidative stress by inducing the generation of ROS^29^. Our previous studies suggest that one possible molecular mechanism involved in ISO-induced MI is the disruption of delicate oxidant/antioxidant balance, which can lead to myocardial injury via oxidative damage^20^,^28^. In the present study, we demonstrated ISO administration induced over-production of ROS in rat and led to myocardial oxidative damage. (Fig. 2A). Lipid peroxidation has been defined as the oxidative deterioration of polyunsaturated lipid. ROS-derived free radicals can react with lipids, if not blocked by sufficient antioxidant molecules, to form lipid peroxides which do extensive damage^30^. Since the major constituents of biological membranes are lipids, their peroxidation can lead to cell damage and death. A significant increase in the levels of lipid peroxidation products (TBARS) in ISO-induced rats appear to be the initial stage to the tissue making it more susceptible to oxidative damage. Over-production of ROS may be responsible for the observed membrane damage as evidenced by the elevated lipid peroxidation in terms of TBARS in the present research. Whereas, myocardial levels of ROS and TBARS were both remarkably down-regulated either by paeonol and danshensu alone or by their combination in our study. Both of paeonol and danshensu are phenolic compounds, and their antioxidant properties are thought to be pharmacologically useful against heart ischemia. Normally, phenolic compounds act by scavenging free radicals^31^ and quenching the lipid peroxidative chain. The hydroxy and phenoxy groups of phenolic compounds donate their electron to the free radicals and quench them. The phenolic radical in turn forms a quinone methide intermediate, which is excreted via the bile^32^. Earlier performed studies has also support the strong efficiency of paeonol and danshensu as the scavenger of different free radicals observed in several biological models^33^,^34^. In this study, levels of ROS and TBARS were remarkably decreased either in paeonol and danshensu alone group or PDSS group as compared with those of ISO-induced rats, which may be due to the powerful antioxidant and free radical scavenging activities. In addition, GSH, a tripeptide which can act as a nonenzymatic antioxidant by reacting with ROS followed by oxidizing GSH to oxidized glutathione (GSSG) and other disulfides, plays an important role in the regulation of variety of cell functions and in cell protection against free radical mediated injury^35^,^36^. Reduction in cellular GSH content and accumulation of GSSG could impair recovery after short period of ischemia. Therefore, the ratio of GSH to GSSG is also considered to be one of sensitive markers of oxidative stress^37^. In this study, we observed significant decrease in GSH/GSSG ratio in the myocardial tissue of ISO-induced rat which is in agreement with several previous studies. Decreased GSH/GSSG ratio might be due to increased utilization of GSH in protecting ‘SH’ containing proteins from lipid peroxides. Interestingly, we found that PDSS markedly increased GSH/GSSG ratio in ISO-induced myocardial tissue (Fig. 2C). Our finding suggests that the combination of paeonol and danshensu could additively attenuate oxidative stress by decreasing levels of ROS and lipid peroxide, thus resulting in less consumption of GSH in ISO-induced myocardial injure rats.

Our investigations in the underlying mechanisms of the cardioprotective effects of PDSS manifested the activation of the Nrf2 pathways. Supramaximal doses of ISO can induce ROS production, which activates diverse signaling pathways by oxidizing cysteine residues on specific target molecules including kinases, phosphatases, and redox-sensitive transcription factors^38^. Redox-sensitive transcription factor nuclear factor erythroid 2-related factor 2 (Nrf2) has been demonstrated to be a critical transcription factor that regulates the induction of phase 2 detoxifying and antioxidant genes^39^. Overexpression of Nrf2 has been proved to eliminate reactive oxidants, whereas impaired Nrf2 activity displayed the opposite effects^40^. Furthermore, the effects of cardioprotective agents such as 4-hydroxy-2-nonenal and MG132 (a proteasome inhibitor), were shown to be abolished in Nrf2-knockout mice^41^, and expression of the downstream target of the Nrf2 signaling pathway, HO-1, was upregulated to act as an important cardioprotective adaptation to counteract pathological left ventricular remodeling in chronic heart failure^42^. In this study, ISO-induced rats exhibited slight increase in Nrf2 activation due to the presence of ROS, which by itself has the capability to activate Nrf2. This result is in accordance with the previous study that mitochondria-derived ROS induce the activation of Nrf2^43^. Significant increase in Keap1 expression was also observed in ISO-induced rats. This indicates that Nrf2 was more associated with Keap1 and thus less translocation of Nrf2 in the nucleus to activate antioxidant gene expression. However, administration of PDSS significantly decreased the Keap1 protein expression along with substantial nuclear accumulation of Nrf2 in rat myocardium. The nuclear factor Nrf2 is bound Keap1 in the cytoplasm under normal conditions. Under oxidative stress or other potentially damaging stimuli, Nrf2 dissociates from Keap1 and translocate into the nucleus, where it binds to antioxidant response element (ARE) sequences, leading to the transcriptional activation of phase II enzymes/antioxidant genes, including HO-1, NQO1, GST and GCL etc.^44^. We further demonstrated that PDSS significantly elevated the expression of HO-1 and activities of NQO1, GST and GCL in ISO-induced myocardial tissue. Our data demonstrates that treatment with PDSS could increase the accumulation of Nrf2 in nucleus, thus promoting the transcription of key antioxidant and phase II enzymes. Nrf2-mediated antioxidant and phase II enzymes may contribute to cellular protection against oxidative stress and to potentiate antioxidant defense capacity in cells.

Multiple lines of evidence demonstrate that apoptosis has been observed in a number of cardiovascular diseases including myocardial infarction, hypertrophy and in heart failure^45^. In our present study, results from TUNEL staining and caspase-3 activity assay showed that administration of ISO produced death of cardiomyocyte in the heart by inducing apoptosis,. PDSS pretreatment significantly prevented the ISO-induced increase in number of TUNEL-positive cells and decrease the activity of apoptotic effector caspase-3, thus protect myocardia from ISO-induced apoptosis. Apoptosis has been shown to perform via the death receptor-dependent pathway (extrinsic pathway) and mitochondria-dependent pathway (intrinsic pathway)^46^. The Fas activating death receptor pathway is critical for cardiac apoptosis, which is easily activated by oxidative stress^47^. Fas, TNF-α and caspase-8 play an important role in regulating the induction of apoptosis in diverse cell types and tissues. Increased level of ROS induced combination of Fas and its receptor FasL leads to the Fas-associated protein with death domain (FADD) and subsequently leading to the activation of caspase-8, which in turn activates caspase-3, leading cell to apoptosis. Caspase-3 is the predominant effector caspases that is sequentially activated, autocatalyzed, and plays a central role in the execution-phase during cell apoptosis. On the other hand, Bcl-2 family proteins and caspases are checkpoints of the apoptosis pathways. The up and downregulation of Bax/Bcl-2 protein is a pivotal factor determining membrane integrity and apoptosis burden. Increased level of ROS induces Bax to permeabilize the external mitochondrial membrane, leading to the release of cytochrome c and the activation of caspases-9 and finally caspases-3. However, this process is blocked by antiapoptotic protein Bcl-2^48^. To explore the effect of PDSS on myocardial apoptosis, we analyzed the expression levels of various proteins related to apoptosis. In the present study, ISO administered rats displayed a reduced levels of myocardial protein expression of Bcl-2 and increased myocardial levels of Bax, cytochrome c, Fas, TNF-α, caspase-3, caspase-8 and caspase-9 when compared to those of normal control rats. We found that PDSS pretreatment inhibited ISO-induced release of cytochrome c from the mitochondria and consequently down regulated the expression of caspase-9, whereas pretreatment with paeonol alone did not affect cytochrome c and caspase-9 expression. Guo *et al.*^49^ have reported that danshensu protects L-02 cells against γ-irradiation-induced injure via inhibition of the caspase-dependent mitochondria apoptosis pathway. Thus it is plausible that the inhibitive effect of PDSS via mitochondria apoptosis pathway maybe totally dependent on danshensu. Simeonova *et al.*^50^ have shown that intracellularly generated ROS can stimulate TNF-α expression in alveolar macrophages. Also, the conformity between the degree of Fas expression and apoptosis increase indicates that change in the expression of Fas was closely related to cardiomyocyte apoptosis^51^. Here, we demonstrated the over-expression of TNF-α, Fas and caspase-8 during myocardial infarction, suggesting that the three aforesaid expressions might play a role in myocardial apoptosis. PDSS administration completely prevented ISO-induced over-expression of TNF-α, Fas and caspase-8. These above findings reveal that paeonol and danshensu have additive effects on myocardial apoptosis. The cardioprotective effect of PDSS might be associated with the modulation of both of mitochondria-dependent pathway and death receptor-dependent pathway.

On the basis of the obtained results that PDSS protected myocardia against oxidative damage, further investigation was performed with focusing on the possible mechanisms involved in the antiapopotic and antioxidative effect of PDSS. The PI3K/Akt pathway is one of the well-documented pathways involved in protection against oxidative stress and plays a critical role in promoting cell survival in the heart^52^. Studies had previously demonstrated that the PI3K/Akt confer powerful cardioprotective effect, when specifically activated at the time of myocardial infarction, provides an amenable pharmacological target for cardioprotection^53^. Akt phosphorylation has been shown to suppress apoptosis and promote cell survival in ischemic heart^54^. Akt regulates cell survival by phosphorylating different substrates that directly or indirectly regulate apoptosis. Akt phosphorylates BAD on Ser136 to promote cell survival by inhibiting its interaction with antiapoptotic Bcl-2 family members like Bcl-xL, thereby further preventing cytochrome c release^55^. Akt also phosphorylates caspase-9 on Ser196, which inhibits its proteolytic activity via a conformation change^56^. In the present study, we found that PDSS significantly up-regulates protein phosphorylation of PI3K and Akt, suggesting that the protective effect of PDSS is due, at least in part, to its ability to upregulate the PI3K/Akt signaling pathway. In addition, there is increasingly evidence to indicate cross talk between the Nrf2 and PI3K/Akt pathways in response to oxidative insults^57^. In our present study, activation and nuclear translocation of Nrf2 by PDSS was observed in the myocardia, whereas it is possibly mediated by two different modes of action. First, PDSS could directly upregulate the level of Nrf2 protein expression and increase Nrf2 nuclear translocation and transcriptional activity, although the mechanism(s) remain unknown regarding how PDSS acts on Keap1 to activate Nrf2/HO-1 pathway. PI3K/Akt pathways are also reported to be involved in the activation of Nrf2-dependent HO-1 expression^58^. Previous studies demonstrated that specific PI3K or Akt inhibitors significantly suppressed the enhanced expression of Nrf2 as well as its cytoprotective effect induced by ADTM, which is a danshensu derivative with a similar structure to that of danshensu, on t-BHP-induced cell injury in H9c2 cells and myocardial injure in rat ischemia model^59^. Therefore, a similar molecular mechanism could be addressed that Nrf2 pathway activation by PDSS is likely associated with PI3K/Akt signaling. Moreover, despite similar increases in Nrf2/HO-1 levels in the paeonol group and danshensu group in the present study, up-regulates protein phosphorylation of PI3K and Akt was not observed in paeonol alone group. Thus it is plausible that Akt activation could not entirely explain the protective effect of PDSS, especially of the role which paeonol plays in the combination. It has been demonstrated that paeonol could protect cell against apoptosis via either ERK or p38MAPK signaling pathways^60^,^61^,^62^. Since ERK and MAPK are also the usual signaling pathways for the upregulation of HO-1 expression^63^,^64^,^65^,^66^. Hence, it is important to discriminate the the crosstalks among different signaling pathways involved in the cardioprocective effect of PDSS against ISO-induced myocardial injure with proper tools, especially including the use of Nrf2-knockout mice and specific inhibitors, in further investigations.

In conclusion, the present study demonstrated that paeonol and danshensu combination exerts significant cardioprotective effects against ISO-induced myocardial infarction in rats. The protective effect is, at least partly, via activation of Nrf2 signaling and involvement of the PI3K/Akt cell survival signaling pathway. The results may partly explain the cardioprotective mechanisms of *Cortex Moutan* and *Radix Salviae Milthiorrhizae* herb pair thus could provide experimental evidence to support the rationality of combinatorial use of traditional Chinese medicine in clinical practice.

## Additional Information

**How to cite this article**: Li, H. *et al.* Paeonol and danshensu combination attenuates apoptosis in myocardial infarcted rats by inhibiting oxidative stress: Roles of Nrf2/HO-1 and PI3K/Akt pathway. *Sci. Rep.* **6**, 23693; doi: 10.1038/srep23693 (2016).

## Supplementary Material

Supplementary Information

## Figures and Tables

**Figure 1:**
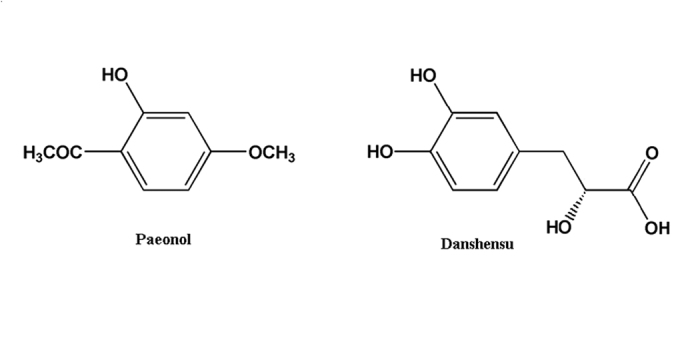
Chemical structures of paeonol (chemical name: 4-methoxy-2-hydroxyacetophenone) and danshensu (chemical name: (2R)-3-(3,4-dihydroxyphenyl)-2-hydroxypropanoic acid).
