# Supplementary material for: Paeonol and danshensu combination attenuates apoptosis in myocardial infarcted rats by inhibiting oxidative stress: Roles of Nrf2/HO-1 and PI3K/Akt pathway
Source: Sci Rep. 2016 Mar 29;6:23693. doi: 10.1038/srep23693 (PMC4810373; doi:10.1038/srep23693)
Supplement: Supplementary Information [file srep23693-s1.doc]

**Paeonol and danshensu combination attenuates apoptosis in myocardial infarcted rats by inhibiting oxidative stress: Roles of Nrf2/HO-1 and PI3K/Akt pathway**

Hua Li ^1, 4, *^, Fan Song ^1, 4, *^, Lin-Rui Duan ^1, 4, *^, Juan-Juan Sheng ^2^, Yan-Hua Xie ^1, 4^, Qian Yang ^1, 4^, Ying Chen ^1, 4^, Qian-Qian Dong ^1, 4^, Bang-Le Zhang ^3^, ^4, @^, Si-Wang Wang ^1, 4, @^

^1^ Department of Natural Medicine, School of Pharmacy, Fourth Military Medical University, 169 West Changle Road, Xi’an 710032, People’s Republic of China

^2^ Department of Aerospace Physiology, School of Aerospace Medicine, Fourth Military Medical University, 169 West Changle Road, Xi'an 710032, People’s Republic of China

^3^ Department of Pharmaceutics, School of Pharmacy, Fourth Military Medical University, 169 West Changle Road, Xi’an 710032, People’s Republic of China

^4^ Collaborative Innovation Center for Chinese Medicine in Qinba Moutains, 169 West Changle Road, Xi’an 710032, People’s Republic of China

^*^ These authors contributed equally to this work.

@ Correspondence and requests for materials should be addressed to S.-W.W. (wangsiw@fmmu.edu.cn) or B.-L.Z. (blezhang@fmmu.edu.cn)

**Table 1S Scoring for histological changes in myocardium of the experimental animals**

| Group(s) | **-** | **+** | **++** | **+++** |
| --- | --- | --- | --- | --- |
| Control | 5 | 0 | 0 | 0 |
| ISO (85 mg/kg, s.c.) | 0 | 0 | 0 | 5 |
| Pae (80 mg/kg, i.g.)+ISO | 0 | 1 | 3 | 1 |
| DSS (160 mg/kg, i.g.)+ISO | 0 | 2 | 3 | 0 |
| PDSS+ISO | 0 | 4 | 1 | 0 |

Photomicrographs were used to evaluate the damage in the heart tissue: (-) no changes; (+) mild changes; (++) moderate changes; (+++) marked changes.

**We use a single membrane instead of the entire membrane to cover each target protein respectively.**

The full-length gel images for Fig. 4A, Fig. 6A, Fig. 6D, Fig. 6H and Fig. 7A

**Fig. 4A Fig. 4D**

**
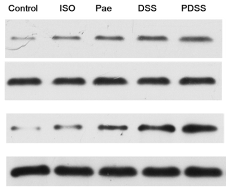
**

**
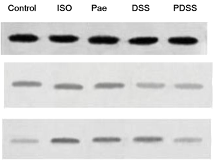
**

**Fig. 6A Fig. 6D**


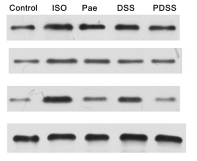

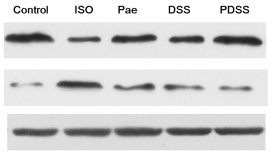


**Fig. 7A**

**
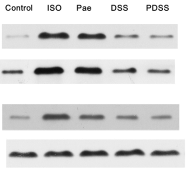

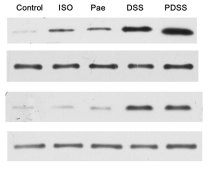
Fig. 6H**
